# Supplementary material for: MicroRNA205: A Key Regulator of Cardiomyocyte Transition from Proliferative to Hypertrophic Growth in the Neonatal Heart
Source: Int J Mol Sci. 2024 Feb 12;25(4):2206. doi: 10.3390/ijms25042206 (PMC10889831; doi:10.3390/ijms25042206)
Supplement: Supplementary file 1 [file ijms-25-02206-s001.zip › ijms-2805276-supplementary.pdf]

Supplemental Table S1. Antibody Information List

Antibody	Vendor	Catalogue Number	Dilution*
CDK1	Santa Cruz	8395	WB: 1/1000
pCDK1	Santa Cruz	136014	WB: 1/1000
Dicer	Cell Signaling	5325	WB: 1/1000
GAPDH	Thermo Fisher	AM4300	WB: 1/1000
Ki67	Cell Signaling	9027	IHC: 1/400
Lats1	Cell Signaling	3477	WB: 1/1000
alphaMHC	Abcam	50967	WB: 1/1000
Mob1	Cell Signaling	13730	WB: 1/1000
pMob1	Cell Signaling	8699	WB: 1/1000
Mst1	Cell Signaling	3682	WB: 1/1000
pH3	Cell Signaling	9701	IHC: 1/200
PTEN	Cell Signaling	9188	WB: 1/1000
pPTEN	Cell Signaling	9551	WB: 1/1000
Rb	Cell Signaling	9309	WB: 1/1000
pRb	Cell Signaling	9307	WB: 1/1000
Sav1	Cell Signaling	13301	WB: 1/1000
Yap	Cell Signaling	14074	WB: 1/1000; IHC 1/200
pYap	Cell Signaling	13008	WB: 1/1000

*WB = Western Blot; IHC = Immunohistochemistry

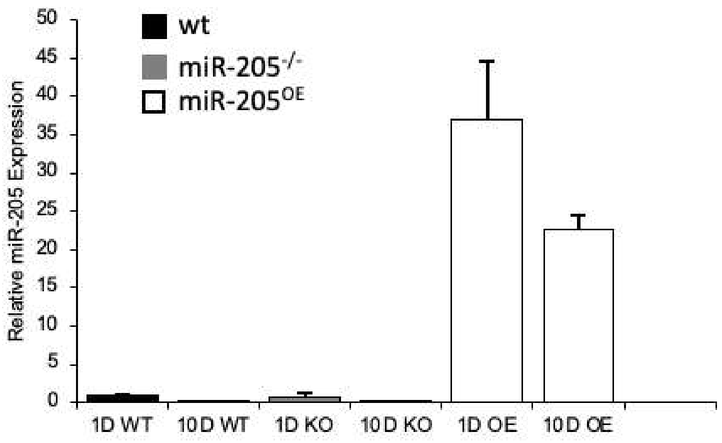

Supplementary Figure S1. RT-qPCR of miR205 confirming the up regulation of miR-205 in cardiac tissue in the cardiac-specific miR-205 over expressing mouse model (n=3 hearts per time point) .
